# How warm is too warm for the life cycle of actinopterygian fishes?

**DOI:** 10.1038/srep11597

**Published:** 2015-07-13

**Authors:** Ryosuke Motani, Peter C. Wainwright

**Affiliations:** 1 Department of Earth and Planetary Sciences, University of California, One Shields Avenue, Davis, California 95616; 2 Department of Evolution and Ecology, University of California, One Shields Avenue, Davis, California 95616

## Abstract

We investigated the highest constant temperature at which actinopterygian fishes can complete their lifecycles, based on an oxygen supply model for cleavage-stage eggs. This stage is one of the most heat-sensitive periods during the lifecycle, likely reflecting the exhaustion of maternally supplied heat shock proteins without new production. The model suggests that average eggs would not develop normally under a constant temperature of about 36 °C or higher. This estimate matches published empirical values derived from laboratory and field observations. Spermatogenesis is more heat sensitive than embryogenesis in fishes, so the threshold may indeed be lower, at about 35 °C, unless actinopterygian fishes evolve heat tolerance during spermatogenesis as in birds. Our model also predicts an inverse relationship between egg size and temperature, and empirical data support this prediction. Therefore, the average egg size, and hence hatching size, is expected to shrink in a greenhouse world but a feeding function prohibits the survival of very small hatchlings, posing a limit to the shrinkage. It was once suggested that a marine animal community may be sustained under temperatures up to about 38 °C, and this value is being used, for example, in paleotemperature reconstruction. A revision of the value is overdue. (199/200)

The exact outcome of the current global warming is uncertain^1^. However, it is likely that Earth’s surface will eventually revisit a greenhouse period, given that there have been quasi-cyclic alterations of green- and ice house periods during the Phanerozoic (last 542 million years), and that the planet is exiting an ice house period^2^. It is therefore important to gauge the impact of a warmer world upon human society and other components of the natural world^1^. For example, there has been a suggestion that the equatorial sea was once lethally hot for fishes during a part of the Early Triassic^3^, following global warming associated with the end-Permian mass extinction about 252 million years ago^2^. If true, Earth’s surface may face similar thermal conditions again, potentially threatening fish resources.

Constancy of Sea Surface Temperature (SST) would also pose a threat. It is known that the thermal tolerance of fishes is higher under fluctuating temperatures than with a constant temperature because of the recovery phase provided during cooler periods^4^,^5^. Modern tropical SST usually has diurnal fluctuation of less than 1 °C^6^, and the difference between the coldest and warmest months is about 2.9 °C when averaging Years 1971 through 2000 based on ORSST^7^. The fluctuation is even smaller when limiting the latitude range to −10° to 10° (Fig. 1A), at about 2.2 °C. A positive correlation is also seen between mean annual SST above 10 °C and the constancy of SST (Fig. 1B). These fluctuations may become even smaller in a greenhouse world, where the latitudinal temperature gradient is expected to be reduced^8^,^9^. To assess the potential impact on fish survival under these greenhouse conditions, it would be useful to know the highest possible upper lethal temperature of fishes over a prolonged duration of a year or more, up to the lifespan of individuals, to bracket the upper limit. However given that even the longest thermal tolerance experiments usually last for several weeks to months, empirical data alone would not allow us to predict such a temperature, motivating the use of mathematical modeling. In the present study, we will focus our attention on actinopterygian fishes given their commercial importance and dominance in marine ecosystems.

## Published Data

Thermal tolerance of fishes has been investigated intensively but most studies are not directly relevant to the current question because they assessed short-term tolerance for less than a few weeks, often followed by a recovery phase under a normal temperature (see Supplementary Note). The resulting lethal temperature estimates would largely inflate our expectation for the thermal tolerance of fishes under the current scenario because the shorter the exposure to heat, the higher the lethal temperature, as in the example discussed below. Another complicating factor is that thermal tolerance of a given fish species fluctuates through its lifecycle. It is known that decreased thermal tolerance occurs during certain stages of development of eggs, embryos, and larvae. Below is a brief summary of what is known of the long-term thermal tolerance of different growth stages of actinopterygian fishes.

### Post-larval Thermal Tolerance

The effect of heat exposure duration is evident in post-larval thermal tolerance. This tendency exists even in estimated Ultimate Upper Incipient Lethal Temperature (UUILT), which is supposed to predict the maximum indefinite survival temperature of fishes—it was shown that UUILT from a 7-day experiments for the Rainbow Trout (*Oncorhynchus mykiss*) was 26 °C when the same from a 60-day experiments was 24.1 °C^10^. The trend is also well illustrated by *Cyprinodon*, which is arguably the best studied genus among heat-tolerant actinopterygians. For shorter-terms, *Cyprinodon variegatus*, which is one of the fishes with the highest thermal tolerance, can survive an exposure to 45.1 °C if the temperature is gradually raised over about 1 hour and then lowered again^11^. However, the tolerance drops to 41.5 °C (50% survival) if it is exposed to a constant temperature for 12 hours^11^. Its highest acclimation temperature (at least 48 hours) is even lower, at about 38 °C, but growth is not observed under this temperature. Truly long-term thermal tolerance of this species is unknown, as with most other species.

Thermal tolerance for growth is even lower than survival temperatures in most fishes. For example, in *Cyprinodon nevadensis nevadensis*, a species closely-related to *C*. *variegatus*, half of the individuals studied ceased their growth at 31 °C and all individuals started to lose body mass by about 34 °C over seven weeks, although fish would still consume food at 36 °C^12^. Similarly, Bull Trout (*Salvelinus confluentus*) stops growing at 13.2 °C although they can survive an exposure to 20.9 °C for 60 days^13^. Fishes of the Persian Gulf do not feed at temperatures higher than 36 °C^14^ despite the occasional presence of such temperature regimes^15^. Indeed, 35 °C is considered to be the threshold value beyond which fish-kill phenomena occur in the Persian Gulf^16^. However, there are exceptions to this general tendency to cease growth at high temperatures. Juveniles of Barramundi (*Lates calcarifer*)^17^ and Nile Tilapia (*Oreochormis niloticus*)^18^ keep growing almost up to their lethal temperatures, to about 39 and 37 °C, respectively, over three weeks, although the growth significantly slows at these temperatures and it is unknown if longer exposure may lead to weight loss. This lack of energy reallocation from growth to body maintenance at high temperatures may have resulted in their absolute thermal tolerances that are lower than those of the most heat-tolerant fishes, such as *Cyprinodon*, at similar body masses.

Thermal tolerance of reproductive behaviors seems to be even lower than for growth. Maximum reproductive temperature for *C. n. nevadensis* is 31 °C under lab conditions^4^,^12^, although it is known to live in a constant-temperature spring at 33 °C^19^. This value, to our knowledge, is the highest constant temperature at which any fish completes its lifecycle in nature, although possibilities remain that they may be able to reproduce at slightly higher temperatures, especially in laboratories.

### Thermal Tolerance of Larvae

The thermal tolerance of fish larvae at hatching is comparable to adult tolerance but reduces greatly toward the end of the yolk-sac stage^20^ and the trend continues toward the stage where notochord flexion occurs^21^. This approximately corresponds to the time of the highest mass-specific oxygen consumption rate^22^,^23^. The total drop in maximally tolerated temperature during this stage amounts to 4 to 10 °C compared to the normal adult tolerance in the fish species examined so far^20^,^21^,^22^,^23^.

### Thermal Tolerance of Eggs

There are two developmental stages where fish eggs are most vulnerable to exposure to a high temperature^24^,^25^. The first is the cleavage stage following fertilization, and the second is a point during gastrulation when mesoderm and ectoderm are differentiated, forming notochord and neural cord^24^. Elevated temperature causes abnormal cell divisions during the first stage, ultimately leading to mortality^24^, whereas the rate of cell division within an individual is affected during the second stage, leading to abnormal differentiation of somite and neural cord^24^. The upper limits for the survival of eggs following short-term exposures in the laboratory were 35.6 °C (34 °C for 50% survival) for *Cyprinodon macularius*^26^, ~36 °C for *C. n. nevadensis*^27^, and 36.4 °C (~36 °C for 50% survival) for *C. rubrofluviatilis*^28^.

The impact on eggs of long-term exposure to elevated temperatures is known only in a limited number of species, where thermal tolerance drops with longer exposure. For example, cleavage-stage eggs of *Pagrus major* may withstand a constant temperature of 31 °C for 7.5 minutes (50% survival) but the tolerance falls to 26.5 °C when exposed to the same temperature for 1440 minutes^25^. Heartbeat stage embryos may withstand 36.5 °C for 7.5 minutes but only 32 °C if exposed for 60 minutes.

It is noteworthy that thermal tolerance during early embryogenesis is similarly low in other vertebrates. For example, the ovarian follicle of mammals is ~1.5 to 2.5 °C cooler than the core body temperature (~36 °C as opposed to ~38 °C in many placentals)^29^. The optimal incubation temperature for bird embryos is said to be between 36 and 39 °C^30^ but the egg temperature is up to 15 °C lower during the first several hours of incubation^30^. Frog embryos do not survive heat shock during the cleavage stage^31^. This is likely related to the lack of heat shock protein production during this stage^31^.

### Thermal Tolerance of Gametogenesis and Spawning

Temperature affects gametogenesis and spawning in many actinopterygian species. Sperm and egg qualities decrease rapidly if the temperature is raised beyond the preferred range of a species^32^,^33^. The highest laboratory record for spawning temperature is known in *Cyprinodon n. nevadensis*, where spawning did not occur in individuals that were kept at 36 °C for 21 days but about half of the individuals kept at 34 °C spawned^4^. Therefore, the highest empirical threshold for successful spawning in actinopterygians is similar to that for egg survival mentioned above, although it may be lower by a degree.

The high temperature sensitivity during gametogenesis, especially spermatogenesis, is found across invertebrates^34^,^35^ and vertebrates^36^, except birds^37^. Its biochemical mechanism is understood best among mammals. In most mammals, spermatogenesis occurs in the scrotum that is about 2 to 7 °C cooler than the core temperature^36^ (i.e., 28.5-.35.5 °C). Data from the mouse revealed that HFS1 (Heat Shock Factor) gene is activated at 35–38 °C in male germ cells as opposed to 42 °C in somatic cells^36^. Phosphorylation of eIF2α (Eukaryotic translation initiation factor) starts at 37 °C in testis, ultimately reducing the availability of the ternary complex and slowing translation initiation to facilitate cell survival for the sake of reduced growth, while possibly also increasing cell apoptosis^36^.

## Summary

The review of empirical observations above suggests that it is difficult to expect any extant fish species to complete its lifecycle under a permanently constant temperature of about 36 °C or higher because no species is expected to survive that temperature during gametogenesis and early embryogenesis. This value assumes that fishes can adjust their egg physiology as did placental mammals, which seem to have the highest incubation temperature of all vertebrates during early embryogenesis. Spermatogenesis is probably more sensitive to heat than early embryogenesis in fishes. Nevertheless, birds have demonstrated that it was possible to raise the thermal tolerance during gametogenesis through evolutionary adaptation, to about 40–41 °C. Thus, the possibility cannot be eliminated that actinopterygians may achieve similarly high heat tolerance during gametogenesis, although it is questionable given that even mammals have not acquired such a trait. If such extreme adaptations as in birds and mammals are impossible, then the threshold temperature could be lower, although it is difficult to determine the exact boundary value based on the data available. The highest empirical temperature known for reproductive cycles of fishes in a natural conditions is 33 °C, and 34 °C in laboratory condition, both in *Cyprinodon* as reviewed above.

## Oxygen Supply Model of Thermal Tolerance

If any of the values listed above represent absolute thresholds physically imposed by the underlying mechanism of thermal tolerance, then we may expect that it is difficult to raise the threshold through evolutionary adaptation. The upper lethal temperature of a species over a prolonged exposure likely represents the temperature at which the increased metabolic oxygen demand from rising temperature exceeds the capacity to supply oxygen to cells^38^, which also increases with temperature but not as fast as metabolic rate^39^. This leads to oxygen shortage, which could be lethal depending on the magnitude and duration. Acclimation to a given temperature would allow a species to raise the lethal temperature to some extent by lowering mitochondrial density and thus slowing metabolic rate^38^.

Given that the cleavage stage is one of the life stages where fishes are most vulnerable to high temperatures, we combined published works to build a simple oxygen supply model for actinopterygian eggs. It describes the maximum egg diameter at which oxygen is supplied to the center of the egg before being completely consumed by metabolism, at a given temperature (see below). The purpose of the model is to test if the egg size is constrained by oxygen availability at incubation temperatures based on a simple set of variables. The model incorporates metabolic rate as a function of temperature and egg size. The metabolic rate of fish eggs of a given size under a given temperature varies within a range, which is implemented as confidence and prediction intervals of the mean prediction. This variability in metabolic rates gives rise to confidence and prediction intervals of the threshold egg diameter estimated from the model. The model also accounts for the temperature dependence of oxygen concentration in water.

### Model description

The model was built to calculate the maximum egg diameter where diffusion can supply oxygen to the center of the egg at a given temperature and basal metabolic rate in a spherical actinopterygian egg, despite the presence of metabolic consumption. Such a diameter can be approximated by an equation^40^:


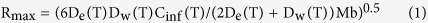


where R_max_, T, D_e_, D_w_, C_inf_, and Mb are maximum egg radius, temperature in K, the oxygen diffusion coefficient in an egg, the oxygen diffusion coefficient in water, the initial oxygen concentration inside the embryo, and basal metabolic rate of the embryo. Note that D_e_, D_w_, and C_inf_ are functions of T, so temperature dependence of dissolved oxygen concentration in liquids is incorporated in the model. It was suggested recently that oxygen dissolved in water becomes easier to extract at higher temperatures despite the decrease in oxygen concentration with rising temperature^39^. The net effect of these two counteracting mechanisms leads to increased oxygen availability to aquatic organisms at higher temperatures, against the traditional view^39^. This effect is not directly incorporated into the present model. However, it does not affect our result because we are trying to find the temperature at which oxygen availability becomes zero, i.e., there is no oxygen dissolved in water regardless of how easy it may be to extract it.

It has been suggested that basal metabolic rate can be approximated by a function of size, temperature, and activation energy for biochemical reactions, of which the last takes a narrow value range of 0.2 to 1.2 eV^41^:


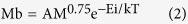


where M, Ei, k, and A are body mass, mean activation energy for biochemical reactions, Boltzmann’s constant, and a constant. Combining the two equations results in:


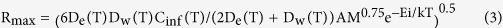


**Figure 2:**
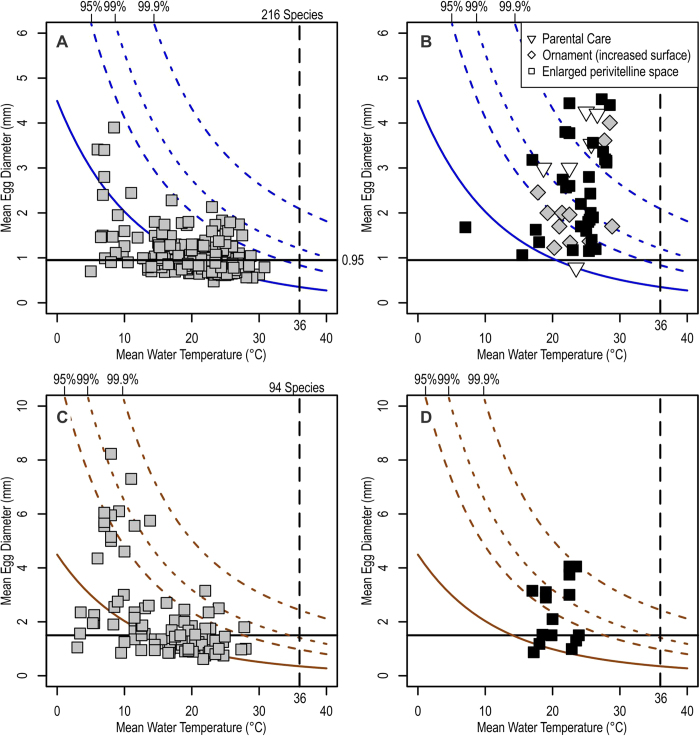
Egg diameters suitable for sufficient oxygen supply under a given water temperature. **A** and **B**, saltwater; **C** and **D**, non-saltwater. Curves represent predictions from eq. 3, with the real line representing the mean prediction, and dashed lines upper prediction intervals with associated percentages. The intervals were derived from the possible range of metabolic rates for eggs of a given size under a given temperature. Thus, species above the mean prediction have metabolic rates that are below average. Empirical data of the egg size and reproduction temperature for fish species are superimposed. **A** and **C**, smooth spherical eggs without parental care; **B** and **D**, other eggs, with either parental care, post-fertilization expansion of perivitelline space, or surface ornamentation. Empirical data were compiled from the literature listed in Supplementary Data.

Using approximations of D_w_ , D_e_, and C_inf_ suggested in the literature^40^, and substituting M with the volume of a sphere (4πR_max_^3^/3) multiplied by the density of the egg (~1.025 g/cc), the combination equations (2) and (3) can be simplified as:


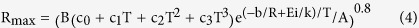


where B = 0.4068, b = 17900, c_0_ = 2.1753, c_1_ = −0.020796, c_2_ = 6.686 × 10^−5^, c_3_ = −7.2074 × 10^−8^, R = 8.134 is the gas constant (all values assume SI units of kg, m, second, and J). Given that there are limits on the capacity for metabolic rates to be modified through adaptation, the model described above allows us to predict the ranges of egg size and temperature that are feasible for adequate oxygen supply. We calculated the confidence and prediction intervals for the metabolic rates of ectothermic vertebrates for a given body mass and temperature based on published data, and converted these intervals using equation (4) to place confidence and prediction intervals for the maximum egg size at a given temperature. Thus, the interval curves in Fig. 2 reflect the possible range of metabolic rate adjustment.

This model has two assumptions that counterbalance each other. First is the placement of the embryo at the center of the egg, when embryos are usually placed around the yolk, not at the exact center. This assumption would lead to underestimation of egg diameter limits. The second is the assumption that the complete loss of oxygen is necessary to affect growth—in reality, a low oxygen concentration would be sufficient to invoke growth failures. This second factor would lead to overestimation of egg diameter limits. Given that the empirical data points are evenly distributed on both sizes of the predicted curve, the two factors seem to be either minor or cancel out each other.

The model may be modified by adding more variables, concerning different life history adaptations. However, our purpose with the model is to test if a simple set of variables can describe the basic patterns even if other factors may add noise to the pattern. Therefore, adding more variables would obscure the question.

### Model versus empirical data

We tested the model in two ways. First, we applied the model (Eq. 4 in Methods) to the Red Sea Bream *Pagrus major*, whose egg mass^42^, egg thermal tolerance^25^, and egg oxygen consumption rates under varying temperature^43^ have been measured. Using the oxygen consumption rate from the late somitogenesis stage, i.e., the last stage before heartbeat starts, leading to violation of the assumption of oxygen supply through only pure diffusion, the model predicted that oxygen becomes unavailable at ~26.5 °C and higher. This threshold value corresponds with the temperature at which the upper thermal tolerance of the egg converges as exposure time becomes very long, toward 1440 minutes^25^. Therefore, the model seems to predict the threshold temperatures well, at least for *P. major*. Further tests are desirable but data under multiple temperatures are not readily available for other species—*P. major* has a high aquaculture interest.

As a second test, we examined if the model matched empirical data of spawning temperature and egg diameters of actinopterygian fishes. The predicted relationship between egg size and the thermal limit for oxygen availability at the center of the egg is depicted in Fig. 2, for spherical eggs in saltwater and non-saltwater. Superimposing mean egg size and reproduction temperature for 216 species of saltwater and 94 species of non-saltwater fishes shows that they are centered around the predicted mean (solid curves in Fig. 2) and fall within 99% prediction intervals of the model (dashed curves in Fig. 2). As stated earlier, the prediction intervals of the diameter are derived from the variability in metabolic rates expected for a given mass and temperature. Thus, those species that appear above the mean prediction curve are expected to have lower metabolic rates than average. Given the even distribution of empirical data around the mean prediction, as well as their position within the statistical prediction interval at 99%, our model seems to pass the empirical test.

**Figure 3:**
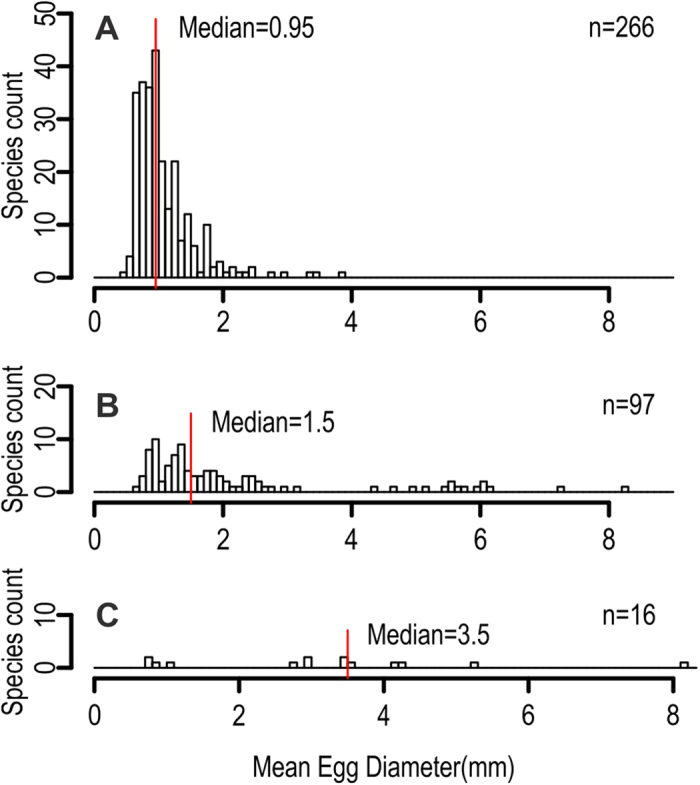
Histograms of the diameter of spherical fish eggs. **A**, smooth saltwater eggs without perivitelline expansion or parental care; **B**, smooth non-saltwater eggs without perivitelline expansion or parental care; **C**, eggs with parental cares. See Supplementary Data for data sources.

The prediction curves in Fig. 2 suggest that smaller eggs can generally tolerate higher temperatures than larger eggs, as expected. The median diameter of the eggs laid in the sea without parental care is about 0.95 mm (Fig. 3A). Such an average egg should have oxygen supply available up to about 36 °C at the 95% prediction interval, i.e., when the metabolic rate is at the predicted minimum for the temperature and egg diameter (Fig. 2A). Freshwater eggs can be larger than their saltwater counterparts (Fig. 3B), because of the higher availability of oxygen in freshwater. Also, flowing waters in rivers elevate oxygen supplies in the absence of parental care, possibly enhancing the departure from the mean prediction in these habitats.

The model assumes an allometric slope of 3/4 for the scaling of metabolism against mass. However, it is possible that a slope of 2/3 may be more appropriate for spherical eggs that exchange gasses through diffusion. We tried this alternative slope and found that it did not affect the major pattern (Supplementary Figure S1), while slightly improving the fit between the model and data by broadening the confidence and prediction intervals. We also tested the model with empirical data for the maximum egg diameters and water temperatures (Supplementary Figure S2) but did not observe a change in the overall pattern. Additionally, we used published lethal temperatures of eggs to test the curves, and again found a match between the two, although the sample size is small given the scarcity of such data (Supplementary Figure S3).

Figure 2 also suggests that fishes are not known to lay eggs at temperatures higher than about 31 °C, despite that oxygen seems to be available at higher temperatures in many cases, and that some fishes inhabit waters that are warmer than 30 °C. The difference may be explained as a safety factor but there may be a second factor other than oxygen supply that limits the temperature tolerance of fish eggs. The most likely candidate is the aforementioned lack of heat-shock protein (hsp) production during the cleavage stages of vertebrate eggs^31^, unlike during the later growth stages. This presumably lowers the heat tolerance of cleavage-stage eggs. Another possibility is that the spawning temperature above 31 °C is not found because sperm quality is lower at higher temperature, as discussed above. Lastly, it is also possible that SST values above 31 °C may be scarce. Note, however, that such seawater temperatures are recorded annually in some parts of the world, such as the Persian Gulf.

## Discussion

**Figure 4:**
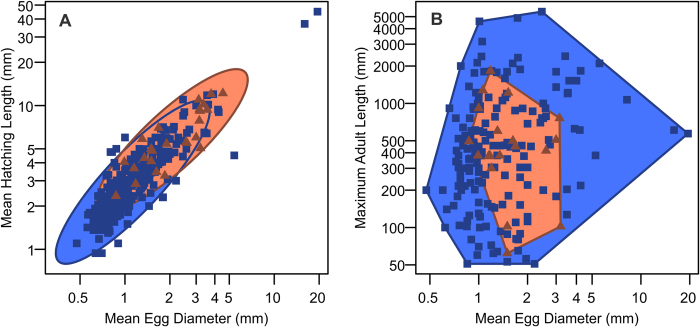
Influence of egg size on later body sizes. **A**, hatching versus egg size, with 95% confidence ellipses; **B**, maximum adult size versus egg diameter, with convex hulls. See Supplementary Data for data sources. Red symbols are for spherical eggs with post-fertilization expansion of perivitelline space, whereas blue symbols are for smooth spherical eggs without such expansion or parental care. Note that species with perivitelline expansion have larger hatching size on average but not necessarily the maximum adult size.

The present study has many implications for fish reproduction under greenhouse conditions. First, the model suggests that the average egg size will shrink with rising mean annual SST, although there is a limit to the shrinkage. The smallest egg diameter recorded for a fish is 0.28 mm in the Shiner Perch (*Cymatogaster aggregata*) but this fish is viviparous^44^ and its small egg yolk does not allow embryos to grow without maternal nutrient supplies. Therefore, the smallest egg size seems to be constrained, at least in part, by the minimum amount of yolk necessary for early growth stages of a minimally small fish embryo. The smallest pelagic egg is about 0.45 mm in diameter (Fig. 2), of the Cupid Wrasse (*Thalassoma cupido*)^45^. Second, if egg size shrinks, the average hatching size of fishes would also decrease because there is a tight correlation between the egg and hatching sizes across fishes (Fig. 4A). Given that suction-feeding is difficult at low Reynolds numbers, and that some fish larvae are already near the threshold value^46^, it is expected that there is little scope for further decreases in hatching size. Third, a similar reduction in the maximum adult size with rising temperature may be expected to some extent, although maximum adult length seems not to be limited by egg diameter unless the egg is smaller than about 1 mm (Fig. 4B).

There are five known ways fish may ameliorate this problem to some extent. First is to abandon spherical egg shape and make eggs elongate, as in many gobiids^47^. Second is to adopt the post-fertilization expansion of perivitelline space, as seen in large Chinese carp species (polyphyletic assemblage within Cyprinidae). Third is to add structures to the egg surface to enhance gas exchanges, as in cyprinodontids^48^. Fourth is to engage in parental care (Fig. 3C), such as fanning, to elevate oxygen availability to eggs. Finally, fifth is the evolution of viviparity. As seen in Fig. 2B and D, it is known that these strategies lead to enlargement of eggs beyond the predicted limits but within a range.

The thermal tolerance of organisms decreases with the level of heat and duration of heat exposure, while SST is expected to be higher and more constant in a greenhouse condition. If the mean annual SST reaches threshold values in a given geographic region, such as the tropics, it would expose actinopterygians to a high temperature that is almost constant for weeks to months. Such may cause the cessation of fish reproduction therein. The threshold temperature is about 36 °C, which seems to be the highest temperature faced by the cleavage-stage eggs of vertebrates, as exemplified by placental mammals. This temperature also coincides with the predicted limit for oxygen supply in average fish eggs. Note, however, that the maximum SST may be a few degrees higher than the mean annual SST, so a mean annual SST lower than 36 °C by a few degrees may already be too warm depending on the spawning season of the species.

A large proportion of marine fishes lay pelagic eggs that drift near the sea surface. These eggs cannot avoid the direct influence of SST. The list of relevant fishes include such commercially important species as many tunas, mackerels, billfishes, flatfishes, snappers, drums, sea basses and sea breams. As the mean annual SST rises in the future, it would likely become important to ensure reproduction of these species.

The threshold value of ~36 °C suggested above for actinopterygian fishes likely has broader taxonomic implications, possibly across vertebrates, given that it is based on mammals and actinopterygians. The oxygen supply model for eggs, however, may not be applicable as broadly. At least lampreys and lungfishes are known to have eggs similar to those of actinopterygians^49^,^50^, and their thermal tolerance patterns closely resemble those of actinopterygians^49^,^50^,^51^. *Latimeria* is viviparous, but given that coelacanths likely were oviparous primitively^52^, the actinopterygians-style oviparity is probably plesiomorphic for Osteichthyes. In contrast, many chondrichthyans and hagfishes have egg cases that are different from actinopterygians eggs^53^,^54^, while other chondrichthyans are viviparous.

The present study has implications for paleotemperature reconstructions. The data for paleotemperature inference are derived from metazoan fossils, including vertebrates, and typically screened based on an assumption that 38 °C is the highest temperature tolerated by marine communities^55^ but the reason is never explained even if citations are traced. If an actinopterygian fossil is present from a given time period and location, it is unlikely that the mean annual SST was higher than about 36 °C at that time and place. Also, when the paleotemperature estimates are based on oxygen isotopes trapped during the growth of conodonts, the same thermal thresholds most likely apply. Conodonts are a group of jawless fossil vertebrates^56^. Extant jawless vertebrates, where known, exhibit patterns of thermal tolerance that are similar to actinopterygian fishes, although none of them are known to live under the high temperatures seen in some actinopterygians, such as *Cyprinodon*^49^,^51^. The oxygen supply model for eggs may or may not apply to conodonts depending on if they had simple actinopterygian-style eggs as in lampreys or egg cases as in hagfishes. Note, however, that egg capsules are typically associated with waters that are cooler than the hottest experienced by actinopterygians eggs^57^. Thus, the model would likely overestimate the thermal tolerance of egg capsules. Then, it is unlikely that conodonts could complete their lifecycle higher than about 36 °C. See Supplementary Note for a discussion of invertebrate heat tolerance.

The aforementioned temperature reconstruction^3^ during the global warming after the end-Permian extinction included an accessory curve that suggested temperatures reaching 40 °C in the late Smithian, whereas the main curve stayed near 36 °C. The accessory temperature curve is highly unlikely because there are late Smithian actinopterygian fossils from Chaohu, Anhui Province, China^58^, which was located around 9° north^59^—the SST difference between −1° to 1° north and 8° to 9° north is minimal for a given longitude in the modern ocean based on ERSST^60^, with a mean difference of less than 0.2 °C between 1910 and 2009. It is often true that SST is higher at 8° to 9° than near the equator. Also, the estimates are based on conodont data. Therefore, we can probably reject the hypothesis that the mean annual SST reached 40 °C in the late Smithian. Nevertheless, equatorial SST was probably almost lethally hot in the Smithian since it was close to the threshold value of 36 °C proposed in this paper.

## Additional Information

**How to cite this article**: Motani, R. and Wainwright, P.C. How warm is too warm for the life cycle of actinopterygian fishes? *Sci. Rep.* **5**, 11597; doi: 10.1038/srep11597 (2015).

## Supplementary Material

Supplementary Information

Supplementary Dataset

## Figures and Tables

**Figure 1:**
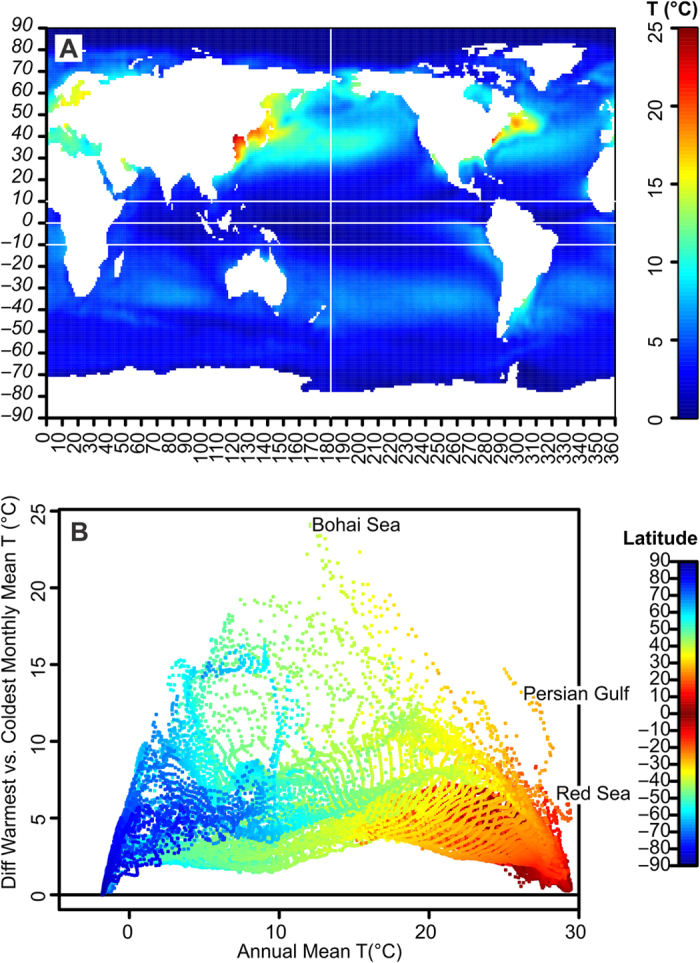
Differences between Sea Surface Temperatures of the warmest and coldest months in the modern oceans. **A**, global map of SST difference distribution. **B**, SST difference relative to the mean annual SST. Colors are the temperature (°C) in A but the latitude (°) in B. Based on long term means between 1971 and 2000 in OISST^7^. Note that annual SST fluctuation is minimal in low latitudes, as well as at very high mean annual SST. This map was plotted by the authors using R, based on a publically accessible data set from NOAA^7^.
