# Supplementary material for: How warm is too warm for the life cycle of actinopterygian fishes?
Source: Sci Rep. 2015 Jul 13;5:11597. doi: 10.1038/srep11597 (PMC4648408; doi:10.1038/srep11597)
Supplement: Supplementary Information [file srep11597-s1.pdf]

Supplementary Information accompanying:

How warm is too warm for the life cycle of actinopterygian fishes?

RYOSUKE MOTANI[1] and PETER C. WAINWRIGHT*[,2]

[1]Department of Earth and Planetary Sciences, University of California, One Shields Avenue, Davis, California 95616.

[2]Department of Evolution and Ecology, University of California, One Shields Avenue, Davis, California 95616.

[*]Corresponding author: Peter Wainwright, Department of Evolution and Ecology, University of California, One Shields Avenue, Davis, California 95616; Email: pcwainwright@ucdavis.edu; Telephone: 530-752-6782.

Supplementary Note

A. Short-term thermal tolerance studies

There is a vast body of literature on the thermal tolerance of fish that cannot be easily covered in a limited space. We therefore focus on a part the literature that are most relevant to our study.

There are at least three different ways in the literature to measure thermal tolerance of organisms, namely CT (Critical Thermal), CL (Chronic Lethal), and LT (Lethal Temperature) methods[1,2]. The observed thermal limits depend on the method as well as exposure time and acclimation temperature[1,2]. CT and CL are dynamic methods, where environmental temperature is changed at a constant rate until the subject organism loses equilibrium or dies. This rate is high in CT (e.g., 0.1 to 1.0 °C/min) and low in CL (e.g., 1 °C/day to 3 °C/month)[3]. It is known that CTM (CT Maximum) is higher than CLM (CL Maximum) because organisms can tolerate short-term exposure to heat better than long-term exposure[3]. LT is a static method, where the subject organism is left under a constant temperature for a preset time span and then usually returned to its normal temperature once the set time period is past and its fate (recovery or death) is observed[4]. Depending on exposure time, LT_{50} (LT at which 50% of the sample survive) may differ greatly[5-7], as in the dynamic methods. For example, Bull Trout (*Salvelinus confluentus*) survived 23.5 °C over 7 days but 20.5 °C for 60 days[7].

The difference between short- and long-term heat exposures is substantial, usually amounting to 4 to 5 °C of average difference in the maximum temperature tolerance[1,5]. Given the context of this study, we are more interested in LT and CL values with long

exposure time than CT values, although CT values are more abundant in the literature given its relative ease of measurement[1,8-15]. Between LT and CL values, the former is preferable given the constancy of temperature.

B. Thermal tolerance in invertebrates

It is worthwhile briefly discussing the long-term heat tolerance of marine invertebrates. A study of 36 tropical invertebrates spanning seven phyla found the gastropod *Echinolittorina malaccana* to have the highest long-term heat tolerance, dying after ~5 weeks of exposure to slowly rising temperatures up to 42 °C[16]: other species died before five weeks. Note, however, that these temperatures are for death points, whereas gastropods first lose normal behavior and then fall into heat coma long before thermal death[17]. It was found that loss of normal behavior and heat coma occurred at ~13 °C and ~6 °C lower than thermal death in 11 species of intertidal mollusks from the UK[17]. The highest tolerance of 46.3 °C (~2hr of exposure) was found in *Melarhaphe neritoides*, but it lost normal behavior at 34 °C and fell into heat coma at 38 °C. It is these lower values that seem most relevant to our study. Under natural conditions, these gastropods experience the highest temperatures only when exposed outside water during the day, and they benefit from cooling during the night that allows physiological recovery. Note that hydrothermal vent polychaetes also live at an average temperature of 30 to 35 °C[18], although they survive short-term exposures (~12 hrs.) of up to 45 °C[19]. Overall, long-term heat tolerance by actinopterygian fishes and invertebrates seem to be similar. Then, paleotemperature data from molluscan fossils probably need to be screen using the same standard proposed for

vertebrate fossils in the present paper. However, detailed investigation of thermal tolerance by the broader metazoans is beyond the scope of the present investigation.

1 Beitinger, T. L., Bennett, W. A. & McCauley, R. W. Temperature tolerances of North American freshwater fishes exposed to dynamic changes in temperature. *Environ Biol Fish* **58**, 237-275 (2000).

2 Bennett, W. A. & Beitinger, T. L. Temperature tolerance of the sheepshead minnow, *Cyprinodon variegatus*. *Copeia*, 77-87 (1997).

3 Fields, R., Lowe, S. S., Kaminski, C., Whitt, G. S. & Philipp, D. P. Critical and Chronic Thermal Maxima of Northern and Florida Largemouth Bass and Their Reciprocal F1 and F2 Hybrids. *T Am Fish Soc* **116**, 856-863, doi:Doi 10.1577/1548-8659(1987)116<856:Cactmo>2.0.Co;2 (1987).

4 Brett, J. R. Temperature tolerance in young Pacific salmon, genus Onchorhynchus. *Journal of the Fisheries Research Board of Canada* **9**, 265-322 (1952).

5 Kikkawa, T. *et al.* Thermal tolerance of small-scale sillago (*Sillago parvisquamis*) during the early developmental stage. *Report of the Marine Ecology Research Institute* **10**, 1-8 (2007).

6 Everich, D. & Gonzalez, J. G. Critical Thermal Maxima of 2 Species of Estuarine Fish. *Mar Biol* **41**, 141-145, doi:Doi 10.1007/Bf00394021 (1977).

7 Selong, J. H., McMahon, T. E., Zale, A. V. & Barrows, F. T. Effect of temperature on growth and survival of bull trout, with application of an improved method for determining thermal tolerance in fishes. *T Am Fish Soc* **130**, 1026-1037, doi:10.1577/1548-8659(2001)130<1026:Eotoga>2.0.Co;2 (2001).

8 Sunday, J. M., Bates, A. E. & Dulvy, N. K. Global analysis of thermal tolerance and latitude in ectotherms. *P Roy Soc B-Biol Sci* **278**, 1823-1830, doi:DOI 10.1098/rspb.2010.1295 (2011).

9 Currie, R. J., Bennett, W. A. & Beitinger, T. L. Critical thermal minima and maxima of three freshwater game-fish species acclimated to constant temperatures. *Environ Biol Fish* **51**, 187-200, doi:Doi 10.1023/A:1007447417546 (1998).

10 Matthews, W. J. & Maness, J. D. Critical Thermal Maxima, Oxygen Tolerances and Success of Cyprinid Fishes in a Southwestern River. *Am Midl Nat* **102**, 374-377, doi:Doi 10.2307/2424665 (1979).

11 Heath, A. G., Turner, B. J. & Davis, W. P. Temperature Preferences and Tolerances of 3 Fish Species Inhabiting Hyperthermal Ponds on Mangrove Islands. *Hydrobiologia* **259**, 47-55, doi:Doi 10.1007/Bf00005964 (1993).

12 Fries, J. N. & Gibson, J. R. Critical Thermal Maxima of Captive Bred Devils River Minnows (Dionda Diaboli). *Southwest Nat* **55**, 544-550, doi:Doi 10.1894/Rje-02.1 (2010).

13 Feminella, J. W. & Matthews, W. J. Intraspecific differences in thermal tolerance of *Etheostoma spectabile* (Agassiz) in constant versus fluctuating environments. *J Fish Biol* **25**, 455-461, doi:10.1111/j.1095-8649.1984.tb04892.x (1984).

14 Fangue, N. A. & Bennett, W. A. Thermal tolerance responses of laboratory-acclimated and seasonally acclimatized Atlantic stingray, Dasyatis sabina. *Copeia*, 315-325 (2003).

15 Darveau, C. A., Taylor, E. B. & Schulte, P. M. Thermal Physiology of Warm-Spring Colonists: Variation among Lake Chub (Cyprinidae: Couesius plumbeus) Populations. *Physiol Biochem Zool* **85**, 607-617, doi:Doi 10.1086/665539 (2012).

16 Nguyen, K. D. T. *et al.* Upper Temperature Limits of Tropical Marine Ectotherms: Global Warming Implications. *PLoS One* **6**, doi:10.1371/journal.pone.0029340 (2011).

17 Evans, R. G. The Lethal Temperatures of Some Common British Littoral Molluscs. *Journal of Animal Ecology* **17**, 165-173, doi:10.2307/1480 (1948).

18 McMullin, E. R., Bergquist, D. C. & Fisher, C. R. Metazoans in Extreme Environments: Adaptations of Hydrothermal Vent and Hydrocarbon Seep Fauna. *Gravitational and Space Biology Bulletin* **13**, 13-23 (2000).

19 Dilly, G. F., Young, C. R., Lane, W. S., Pangilinan, J. & Girguis, P. R. Exploring the limit of metazoan thermal tolerance via comparative proteomics: thermally induced changes in protein abundance by two hydrothermal vent polychaetes. *P Roy Soc B-Biol Sci* **279**, 3347-3356, doi:10.1098/rspb.2012.0098 (2012).

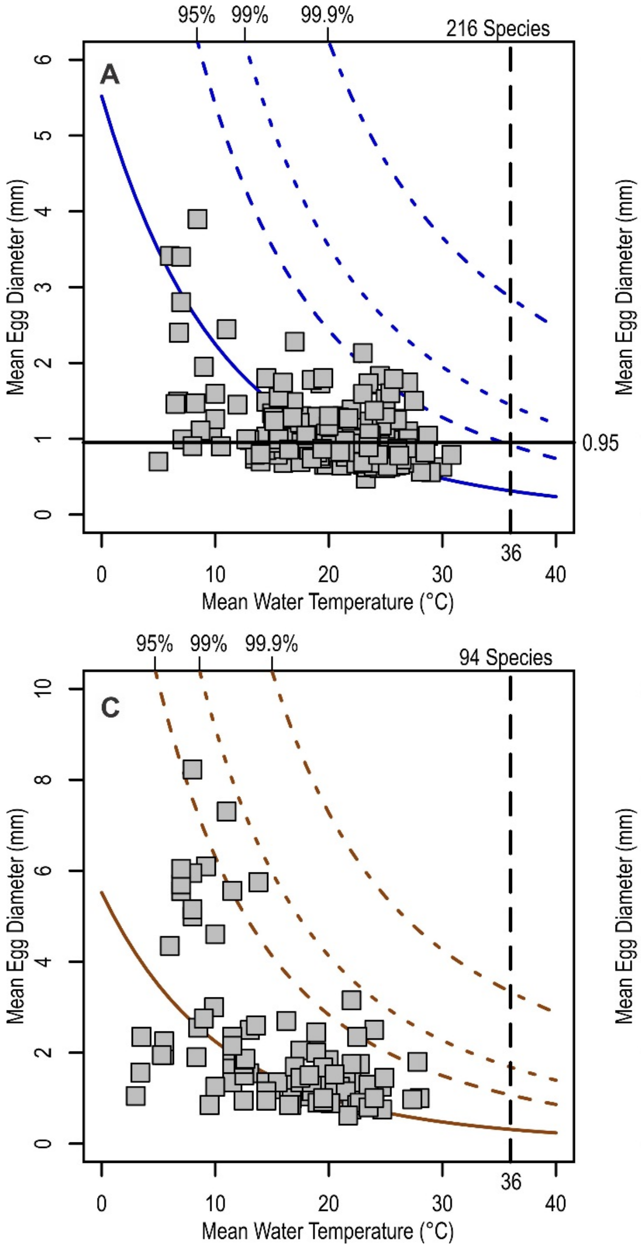

Supplementary Figure S1. Same as Fig. 2 but with a metabolic slope of 2/3. See Fig. 2 caption for details.

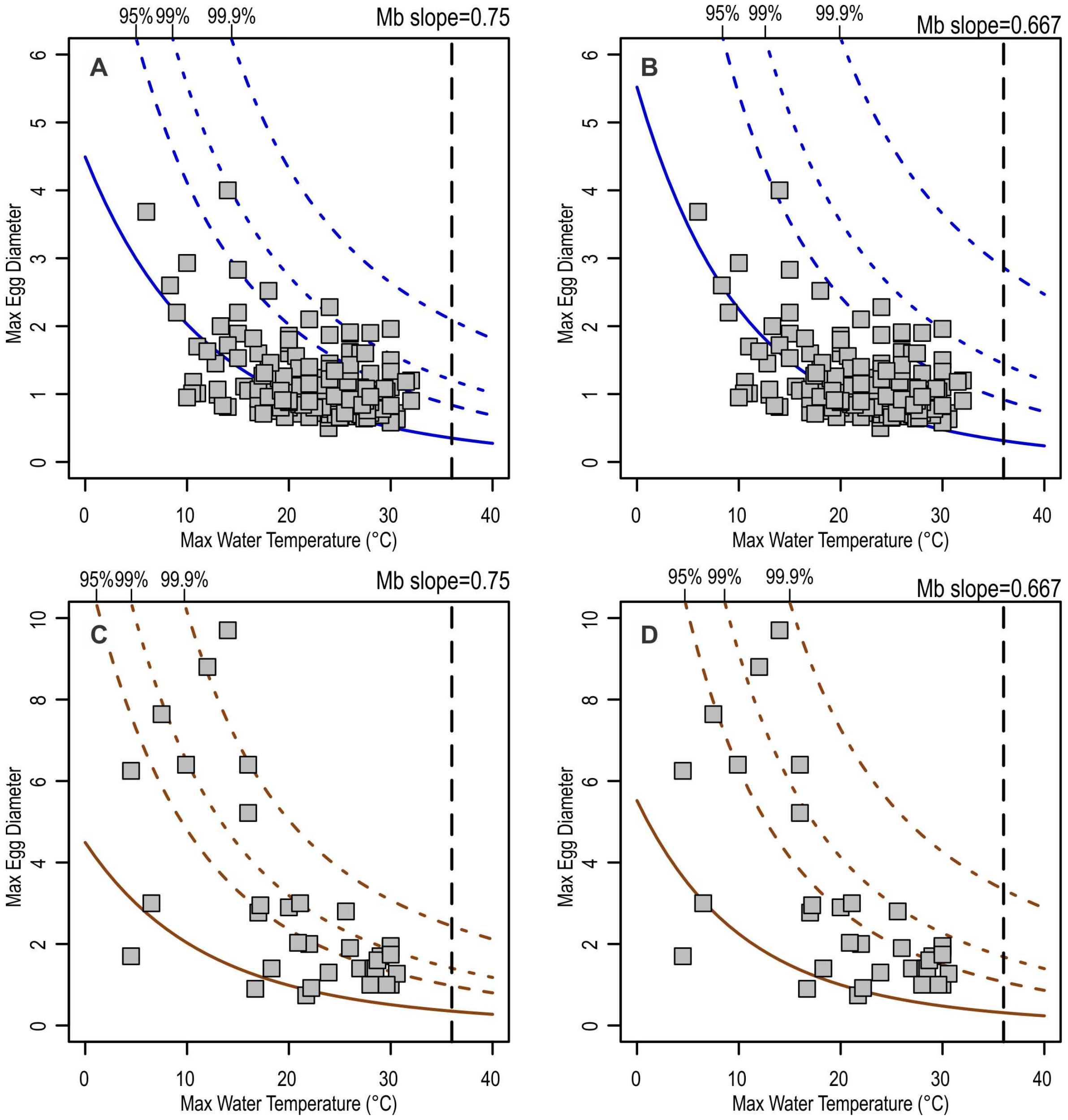

Supplementary Figure S2. Same as Fig. 2 and Supplementary Fig. S1 but with maximum values of egg diameter and water temperature for empirical data. The combination of maximum egg diameter and temperature is unrealistic given that eggs laid at the maximum temperature are expected to be minimally small. Therefore, this plot is only given to

facilitate a comparison. A, B, saltwater; C, D, non-salt water. A and C are based on a metabolic slope of 3/4, while B and D assumes a slope of 2/3.

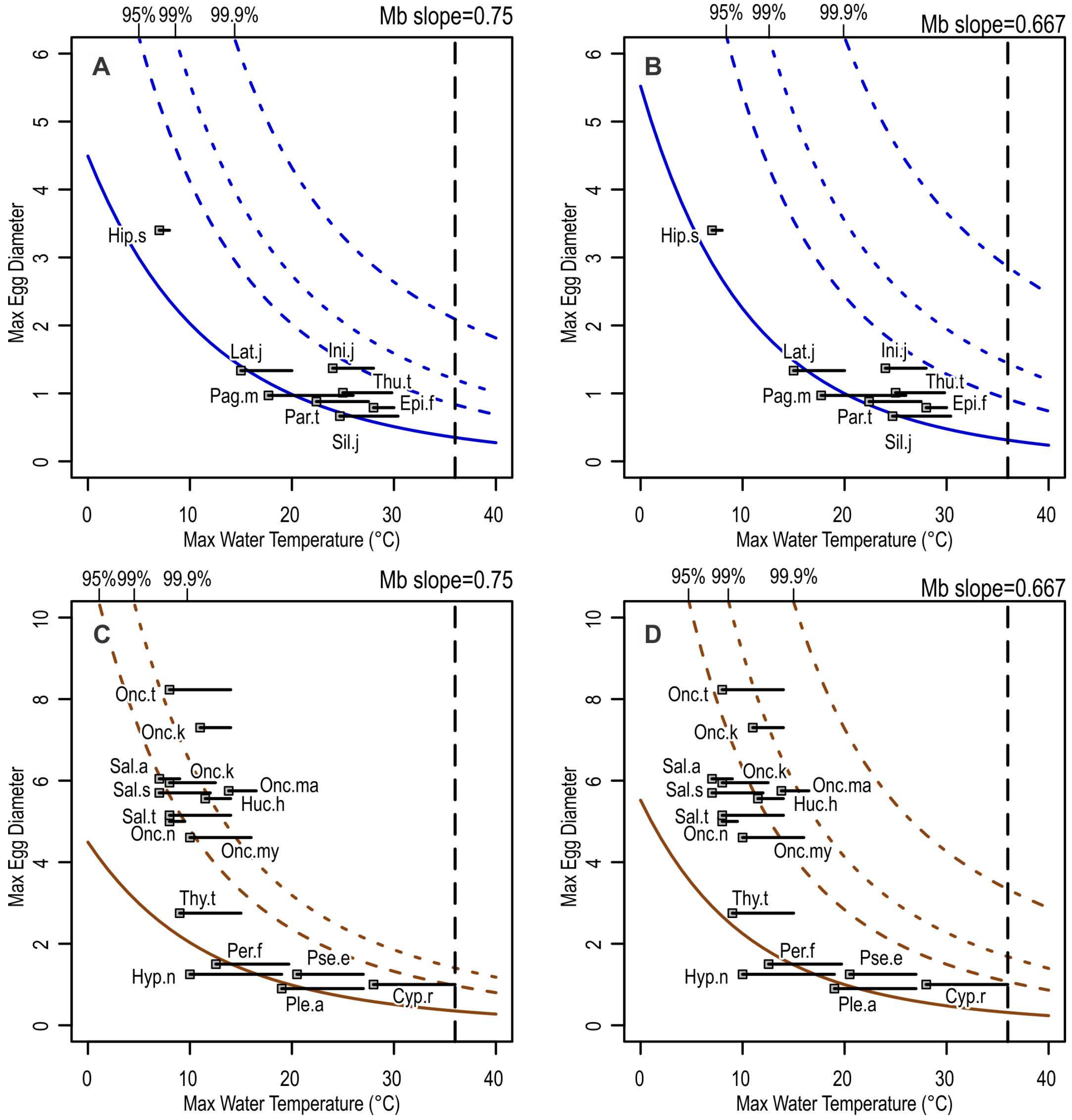

Supplementary Figure S3. Same as Supplementary Fig. S2 but with 50% lethal temperature ranges listed in Supplementary Data. See Supplementary Data for species names.
